# Profile of Cytokines Associated with SARS-CoV2 Seropositivity in Multiple Sclerosis Patients and Its Persistence over Six Months

**DOI:** 10.3390/jcm14113736

**Published:** 2025-05-26

**Authors:** Agustín Sancho-Saldaña, Anna Gil-Sánchez, Bibiana Quirant-Sánchez, Marc Boigues, Marc Canudes, Silvia Peralta, María José Solana, Cristina González-Mingot, Laura Quibus, Eva Martínez-Cáceres, Pascual Torres, José Vicente Hervás, Judith Moreno-Magallon, Luis Brieva

**Affiliations:** 1 Servicio de Neurología, Hospital Universitario Arnau de Vilanova, 25198 Lleida, Spain; asanchos.lleida.ics@gencat.cat (A.S.-S.); speralta.lleida.ics@gencat.cat (S.P.); mjsolana.lleida.ics@gencat.cat (M.J.S.); cgonzalezm.lleida.ics@gencat.cat (C.G.-M.); 2 Servicio de Neuroinmunología, Institut de Recerca Biomèdica de Lleida-IRBLleida, 25198 Lleida, Spain; agil@irblleida.cat (A.G.-S.); mcanudes@irblleida.cat (M.C.); lquibus.lleida.ics@gencat.cat (L.Q.); pascual.torres@udl.cat (P.T.); jmoreno@irblleida.cat (J.M.-M.); 3 Immunology Division, Germans Trias i Pujol University Hospital and Research Institute, Hospital Universitari Germans Trias i Pujol, 08916 Badalona, Spain; bquirant.germanstrias@gencat.cat (B.Q.-S.); mboigues.germanstrias@gencat.cat (M.B.); emmartinez.germanstrias@gencat.cat (E.M.-C.); 4 Department of Cell Biology, Physiology, Immunology, Autonomous University of Barcelona, 08193 Cerdanyola del Valles, Spain; 5 Department of Medicine, University of Lleida (UdL), 25198 Lleida, Spain; 6 Metabolic Pathophysiology Research Group, Department of Experimental Medicine, Lleida Biomedical Research Institute (IRBLleida), University of Lleida (UdL), 25198 Lleida, Spain; 7 Servicio de Neurología, Hospital de Sant Joan Despí Moisés Broggi, 08970 Barcelona, Spain; jvherbasg@csi.cat

**Keywords:** multiple sclerosis, SARS-CoV-2, cytokines, seropositivity, IL-18, disease-modifying therapies

## Abstract

**Background:** Patients with multiple sclerosis (pwMS) receiving disease-modifying therapies (DMTs) may exhibit altered immune responses to infections such as SARS-CoV-2. This study aimed to characterize the cytokine profiles associated with prior SARS-CoV-2 infection and to identify immune markers related to the persistence of the humoral response in pwMS. **Methods:** A total of 90 pwMS were recruited before the introduction of COVID-19 vaccination in Spain; 46 were seropositive—defined by the presence of IgG, IgM, or IgA antibodies against SARS-CoV-2—and 44 were seronegative. We compared baseline cytokine levels between groups and followed seropositive individuals for six months to assess IgG antibody persistence. **Results:** Seropositive patients showed significantly lower baseline levels of IL-10, IL-23, and IFN-α compared to seronegative individuals. Notably, elevated IL-18 at baseline was associated with persistent IgG seropositivity at six months. **Conclusions:** These findings suggest a distinct cytokine profile in SARS-CoV-2–exposed pwMS and highlight IL-18 as a potential marker of sustained humoral response. This study provides insight into host-virus immune dynamics in MS patients and may help guide future strategies for infection monitoring and immune evaluation in this population.

## 1. Introduction

Cytokines are soluble pleotropic proteins with low molecular weight that regulate and oversee inflammatory response via complex networks [1,2]. Cytokines are secreted by different cell types (leukocytes, monocytes, endothelial cells and macrophages) and can act on several cell types, inducing multiple biological activities [3]. These soluble factors play a pivotal role in orchestrating the immune response, acting as crucial mediators within the intricate communication network of the immune system. Their essential functions encompass activation, differentiation, proliferation, maturation, and migration of immune cells, allowing for precise control over cell behaviour during immunological and inflammatory responses via paracrine and autocrine mechanisms and, thereby influencing overall health [2,4]. Consequently, the levels of cytokines are recognized as an essential indicator for evaluating clinical disorders and may serve as biomarkers for many diseases [2]. They play a pivotal role in triggering and modulating the immune responses in Multiple Sclerosis (MS) [4,5]. Their involvement is also critical in immune defense mechanisms against infectious agents, highlighting their dual importance in both autoimmune pathogenesis and host protection.

MS is a chronic immune-mediated disease characterized by inflammation, demyelination, gliosis, and neurodegeneration in the central nervous system (CNS) [6]. This demyelination and injury observed in MS can be triggered by cytokines, which facilitate the breakdown of the blood-brain barrier and the recruitment of immune cells into the central nervous system, culminating in a broad array of symptoms [4]. Pro-inflammatory cytokines, including interleukin (IL)-17, IL-22, tumour necrosis factor-α (TNF-α), IFN-γ, IL-1, IL-12, and GM-CSF, drive inflammation, disrupt the blood-brain barrier, and promote demyelination and axonal degeneration. IL-17 and IL-22 are particularly significant for CNS inflammation and astrocytic pathology, while TNF-α and IFN-γ have complex roles, influencing both inflammatory progression and immune modulation. Conversely, anti-inflammatory cytokines such as IL-10, IL-4, and IFN-β1 provide protective effects by reducing inflammatory responses, promoting Th2 differentiation, and enhancing immune regulation. The interplay between these cytokines underscores the immunopathological complexity of MS [7].

On the other hand, it is well known that “cytokine storm syndrome” is responsible for the poor prognosis of critical Corona Virus Disease 2019 (COVID-19) cases [8,9]. Following infiltration into respiratory epithelial cells, severe acute respiratory syndrome coronavirus 2 (SARS-CoV-2) triggers an immune response through the activation of pathogenic Th1 cells and intermediate CD14+CD16+ monocytes. SARS-CoV-2 infection is accompanied by activation of immune-inflammatory pathways including increased levels of IL-1β, IL-6, IL-7, IL-18, IL-10, TNF-α and granulocyte-colony stimulating factor (G-CSF). This is accompanied by a weak IFN-I response, which may be an important amplifier of cytokine production [8,10]. In addition, COVID-19 may be influenced by IFN- γ or its precursors, such as interleukin IL-18, IL-12, or IL-23 [11].

During the COVID-19 pandemic, pwMS faced unique challenges arising from the interplay between SARS-CoV-2, the underlying immune-mediated disease, and the use of DMTs. Key concerns included the potential for increased susceptibility to SARS-CoV-2 in pwMS due to the complex interactions between the virus and the immune system and the immunomodulatory or immunosuppressive effects of DMTs; or the potential association between SARS-CoV-2 infection and an elevated risk of demyelination. Furthermore, in later stages of the pandemic, questions emerged regarding the adequacy of humoral and cellular immune responses following natural SARS-CoV-2 infection or vaccination [12]. These considerations underscore the ongoing importance of investigating the immune response to SARS-CoV-2 in pwMS.

Our study aims to evaluate the presence of a cytokine profile associated with SARS-CoV-2 seropositivity in pwMS and to assess whether a specific cytokine profile correlates with the persistence of seropositivity at six months.

## 2. Materials and Methods

### 2.1. Study Design and Patients

This multi-center, prospective observational study was conducted in two parts.

In the first part, we enrolled pwMS who were receiving any form of disease-modifying therapy (DMT) and who tested positive for IgG, IgM, or IgA antibodies against SARS-CoV-2 prior to the initiation of COVID-19 vaccination in Spain, as part of a scheduled seroprevalence protocol, regardless of whether the patients were asymptomatic or not We manually matched a control group of pwMS who were seronegative for all SARS-CoV-2 antibodies within the same timeframe. To ensure comparability, we applied a statistical homogeneity test to assess the baseline characteristics between the two groups (basal seropositive and basal seronegative). We then conducted a comparative statistical analysis of cytokine profiles, including IL-1, interferon-alpha (IFN-α), IL-6, TNF-α, interferon-gamma (IFN-γ), monocyte Chemoattractant Protein-1 (MCP-1), IL-8, IL-10, IL-12, IL-17, IL-18, IL-23, and IL-33, across both groups.

In the second part, we followed the SARS-CoV-2 seropositive pwMS group prospectively for six months to reassess seropositivity to IgG SARS-CoV-2 antibodies. Two patients were excluded due to lack of information about serological status at six months. At the six-month follow-up, we performed a comparative statistical analysis of the baseline cytokine profiles between patients who remained seropositive and those who had become seronegative 6 months later.

### 2.2. Blood Samples for Antibodies and Cytokine Analysis

Peripheral blood samples, both at baseline and at 6-month follow-up, were taken between March 2020 and September 2020, before the start of COVID-19 vaccination in Spain (28 December 2020). The samples obtained were centrifuged and the serum frozen at −80 °C. SARS-CoV-2 antibodies (IgG, IgM, and IgA anti-SARS-CoV-2) were analysed using an enzyme-linked immuno-sorbent assay (ELISA) containing a pool of S and N recombinant antigens (Diapro^®^, Sesto San Giovanni, Italy), according to the manufacturer’s instructions. In addition, those sera-positive for IgG anti-SARS-CoV-2 antibodies were re-analysed with an independent confirmatory ELISA assay that separately measured IgG antibodies to spike glycoprotein-1 (S1), spike glycoprotein-2 (S2), or N antigens. Results were expressed with an index value, calculated as the ratio between the optical density (OD) of each sample and the OD of the cut-off reagent provided by the manufacturer. Index values ≥1.1 were considered positive and <1.1 were classified as negative.

The cytokines were measured using the LEGENDplex ™ Human Inflammation Panel 1, with reagents and analysis software from BioLegend (San Diego, CA, USA), and the samples were acquired using a FACS Fortessa cytometer from Becton Dickinson (Franklin Lakes, NJ, USA), as described in the kit protocol. Briefly, for each sample 25 μL of serum were incubated with 25 μL of a mix of anti-cytokine antibody-coated beads. Then the beads were incubated with 25 μL of the secondary antibody and 25 μL of Streptavidin-Phycoerythrin consecutively. Finally, the beads were acquired through the FACS Fortessa BD^®^ flow cytometer. For each type of cytokine, a 5PL standard curve was performed and was used to calculate de cytokine concentration in the samples.

### 2.3. Statistical Analysis

Data are presented as absolute and relative frequencies for categorical variables, and as median and interquartile range (IQR) for quantitative variables.

Differences in basal characteristics between seropositive and seronegative groups are evaluated through paired *t*-test or Wilcoxon signed-rank test (numerical variables) and McNemar test (categorical variables).

For comparing cytokines levels between groups, the Wilcoxon signed-rank test was applied. The rank biserial correlation was estimated as the measure of effect, which takes values ranging from −1 to 1.

To evaluate the effect of cytokine levels on serological status (positive versus negative) Generalized Linear Mixed Models [13] were applied. The model includes as covariates those basal variables showing differences between the groups. The odds ratio (OR) along with its 95% confidence interval was estimated. Cytokine levels were log transformed for ensuring normality in the residuals. Since the reduced sample size, one model is estimated for each of the cytokines.

The Alpha Level Was Set at 0.05 for All the Analysis.

The free software R (version 4.3.1; R Core Team, Vienna, Austria) was used for the statistical analysis. The lme4 package (version 1.1-34; Bates et al. [14], R Foundation for Statistical Computing, Vienna, Austria) was used for estimating the Generalized Linear Mixed Models (GLMM). The package is maintained by contributors including Douglas Bates [14], and is distributed via The Comprehensive R Archive Network (CRAN), managed by the R Foundation for Statistical Computing, Vienna, Austria.

### 2.4. Ethics

This study was subject to thorough evaluation and approval by the Ethics Committee, as detailed below:

Ethics Committee Name: Clinical Research Ethics Committee of Arnau de Vilanova

University Hospital in Lleida.

Approval Code: CEIC-2253.

Approval Date: 16 April 2020.

### 2.5. Use of IA

ChatGPT4 was used solely for minor English language refinement and grammatical corrections of some sections of the manuscript.

## 3. Results

A total of 90 pwMS were included in the study, of whom 46 were seropositive—defined as having IgG, IgM, or IgA antibodies against SARS-CoV-2—and 44 were seronegative. The median age was 47 years (IQR 43–53), with a predominance of females (74.4%, 67/90). Regarding MS phenotype, 78 patients (86.7%) had relapsing-remitting MS (RRMS), while 12 (13.3%) had secondary progressive MS (SPMS). The median duration since symptom onset was 13 years (IQR 8–20), and the median Expanded Disability Status Scale (EDSS) score was 1.5 (IQR 0–3.8). Only 2 out of 46 seropositive patients experienced severe or moderate symptoms of SARS-CoV-2, while the remaining 44 were either asymptomatic or had mild symptoms. First-line disease-modifying therapies (DMTs)—including interferon, teriflunomide, and dimethyl fumarate—were used by 46 patients (51.1%), whereas second-line DMTs—such as cladribine, fingolimod, alemtuzumab, natalizumab, ocrelizumab, or rituximab—were used by 44 patients (48.9%). Detailed baseline characteristics and treatment regimens are provided in Table 1.

The median duration of anti-CD20 treatment (Rituximab or Ocrelizumab) was 6 months (IQR 3.25–12). When comparing duration by serostatus, we observed no significant difference:6 months (IQR 5–9) in seropositive vs. 4 months (IQR 2–25.5) in seronegative, *p* = 0.71. The full data—median durations for each DMT and their statistical comparisons with serostatus—have been included in the Supplementary Material (Supplementary Table S1).

A homogeneity test revealed that the two groups (basal seropositive and basal seronegative) were comparable across all variables, except for “time duration since symptom onset”, which was significantly shorter in the seronegative group.

Median levels of the cytokines IL-23 (3.8 vs. 7.9, *p* = 0.025) and IL-10 (5.5 vs. 6.9, *p* = 0.049) were significantly lower in the basal seropositive group, with a moderate effect size (r = 0.39 and r = 0.34, respectively). A detailed comparison of cytokine levels between the two groups, assessed using the Wilcoxon signed-rank test, is shown in Table 2.

**Figure 1:**
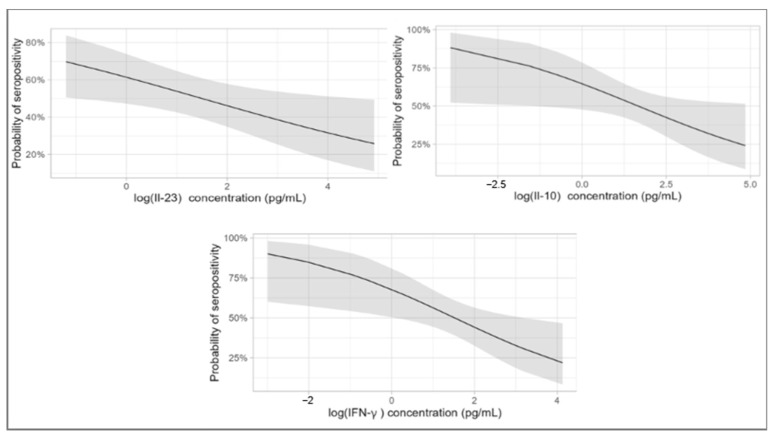
Graphical representation of the mixed logistic regression models illustrating the probability of seropositivity based on cytokine levels of IL-23, IL 10 and IFN-γ.

Results from the mixed logistic regression models showed significantly lower levels of IL-23 (OR 0.734 (95% CI = 0.564; 0.956)), Il-10 (OR 0.697 (95% CI = 0.495; 0.982)) and IFN- γ (95% CI = OR 0.613 (0.414; 0.908)) in the seropositive group (Figure 1). Time duration since symptom onset as well as lymphocytes levels were included as covariates. The full mixed logistic regression model has been included in the Supplementary Material (Supplementary Table S2).

**Figure 2:**
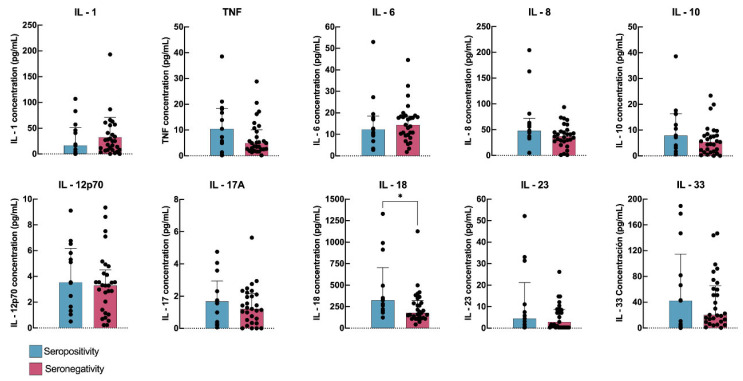
Graphical representation of the serological status at 6 months according with cytokine levels at basal moment. * Statistically significant.

Of the 42 basal seropositive patients who were followed up at 6 months, 13 remained seropositive, while 29 became seronegative. Elevated levels of IL-18 were significantly associated with persistent IgG seropositivity at 6 months (*p* = 0.020), as detailed in Table 3 and Figure 2.

## 4. Discussion

Extensive literature has investigated the role of cytokines in SARS-CoV-2 infection and in the pathogenesis of multiple sclerosis (MS) independently. However, few studies have specifically investigated the cytokine profile of people with MS (pwMS) affected by SARS-CoV-2 and its relationship with the humoral immune response during follow-up.

Cytokine storm syndrome, characterized by the excessive production of pro-inflammatory cytokines such as IL-6, IL-8, and TNF-α, has been proposed as a key driver of inflammation in the pathogenesis of severe COVID-19 [15]. However, it has been shown that the cytokine profile in SARS-CoV-2 infection varies across the entire spectrum of disease severity, from mild to severe cases, and undergoes temporal changes during the recovery phase [16,17].

A study analysing the immune response against SARS-CoV-2 in asymptomatic patients reported significantly lower levels of Th1-associated cytokines, including IFN-γ, IL-1β, and IL-18, as well as reduced systemic levels of the Th2 cytokine IL-5 [18]. Another study found no significant differences in cytokine levels between asymptomatic patients and healthy controls [19]. Significant elevations in IFN-α, TNF-α, and GM-CSF levels were observed in recently symptomatic patients, while increased levels of IFN-α and TNF-α were also significant in previously symptomatic groups [19].

On the other hand, pro-inflammatory cytokines such as IL-6, IL-1β, IL-17, IL-22, TNF-α, IL-12, and IFN-γ are thought to play a pivotal role in the pathogenesis of multiple sclerosis (MS) through various signaling pathways. Conversely, reduced levels of anti-inflammatory cytokines like IL-4 and IL-10 suggest an imbalance between Th1 and Th2 immune responses [4,7,20].

In this study, we observed lower levels of the cytokines IL-23, IL-10, and IFN- γ in seropositive pwMS compared to basal seronegative patients.

IL-23 induces neuroinflammation and contributes to tissue damage by promoting the differentiation of Th17 cells leading to a proinflammatory state [4]. Given the involvement of Th17 cells in severe immune injury during SARS-CoV-2 infection, IL-23 inhibitors have been proposed as potentially beneficial in reducing the severity of COVID-19 symptoms [21].

IL-10, on the other hand, inhibits neuroinflammation and promotes neuroprotection and repair by reducing the production of pro-inflammatory cytokines and enhancing the differentiation of regulatory T cells [22]. IL-10 can function as an endogenous danger signal, released by damaged tissues in an attempt to protect the organism from harmful hyperinflammation, such as SARS-CoV-2 [23].

In this sense, elevated levels of IL-23, IL-10, and TNF-α have been associated with a worse prognosis in SARS-CoV-2 infection [24].

In our study, however, because all patients were either asymptomatic or paucisymptomatic, we expected a lower pro-inflammatory IL-23 response to SARS-CoV-2, accompanied by a reduced IL-10 response. This is likely because the protective release of IL-10 from potentially damaged tissues was not necessary in the absence of significant tissue injury or severe inflammation.

IFN-γ, the sole member of type II interferons (IFN-II), is predominantly produced by T cells and natural killer (NK) cells upon stimulation with antigens and cytokines [25,26]. IFN-γ plays a pivotal role in stimulating antigen-specific adaptive immunity and activating innate immunity, particularly through macrophage activation. However, SARS-CoV-2 evades host IFN responses by escaping immune recognition and interfering with antigen presentation [27]. Moreover, the depletion of IFN-γ-producing T cells and NK cells observed during SARS-CoV-2 infection may plausibly account for the reduced plasma IFN-γ levels [25].

Additionally, certain DMTs are known to exert direct effects on cytokine-mediated pathways and neuroinflammation within the central nervous system [28]. However, in our study, the baseline groups were homogeneous with respect to the distribution of DMTs.

To date, only one study has analysed the cytokine profile in people with MS (pwMS) with and without known SARS-CoV-2 infection. The study suggests an imbalance in circulating Th1/Th2 cytokines, characterized by elevated levels of pro-inflammatory cytokines such as TNF-α and IFN-γ, and reduced levels of anti-inflammatory cytokines like IL-4, in pwMS compared to healthy controls. This cytokine imbalance was found to be even more pronounced after SARS-CoV-2 infection [29]. In this study, however, pwMS on immunosuppressive medication were excluded, which could explain in part the discrepancy with our results.

In addition, we report that elevated levels of IL-18 at baseline were associated with the persistence of SARS-CoV-2 seropositivity at 6 months.

IL-18 is a potent pro-inflammatory cytokine involved in host defense against infections and the regulation of both innate and adaptive immune responses. Due to its critical role in immunity, IL-18 is implicated in several autoimmune diseases, such as psoriasis, systemic lupus erythematosus MS, as well as in infectious diseases like SARS-CoV-2 [30]. In the case of the latter, elevated IL-18 levels have also been associated with poor outcomes following infection [31,32]. Markedly elevated serum IL-18 levels are associated with severe disease and mortality in some viral infections characterized by cytokine storms.

Due to its role in the humoral adaptive response, we think pwMS with higher IL-18 response in contact with SARS-CoV-2 could have reinforced the persistence of humoral response at 6 months. In fact, in animal models, Il-18 has been successfully tested for potentiation humoral response to vaccine antigens [33]. Although IL-18 primarily drives Th1 immune responses, it is well-established that, depending on the cytokine constellation, IL-18 can also promote Th2-type responses and facilitate antibody production [33,34].

One of the key strengths of our study is that it was conducted prior to the initiation of COVID-19 vaccination, avoiding potential immune system interference and providing valuable insights relevant to future pandemics.

Our study has several limitations. First, the small sample size limits the power to detect differences between groups. Second, we did not include a healthy control group for comparison. Additionally, we did not analyse the full spectrum of cytokines potentially involved in the SARS-CoV-2 response and MS pathogenesis. Lastly, we did not perform a quantitative analysis of antibody levels, which could have provided more detailed information, particularly regarding the relationship between antibody titers and their persistence at 6 months. Additionally, our follow-up period was relatively short (6 months); however, the study was not extended over time to prevent interference from the initiation of SARS-CoV-2 vaccination programs that began in late 2020.

## 5. Conclusions

In conclusion, our findings identified a distinct cytokine profile in pwMS infected with SARS-CoV-2, characterized by reduced levels of IL-23, IL-10, and IFN-α. Additionally, elevated IL-18 was associated with the persistence of SARS-CoV-2 antibodies at six months, suggesting a more robust humoral response in these patients. Future studies should prioritize longitudinal cytokine monitoring and mechanistic analyses to disentangle protective versus detrimental effects of these immune shifts.

## Figures and Tables

**Table 1:** Baseline characteristics and DMTs *.

	*n* = 90
Sex	
Female	67 (74.44%)
Male	23 (25.56%)
Age (years)	
Median [IQR]	47.00 [43.00; 53.00]
MS type	
Relapsing remitting MS (RRMS)	78 (86.67%)
Secondary progressive MS (SPMS)	12 (13.33%)
EDSS	
Median [IQR]	1.50 [0.00; 3.88]
Time since symtomps onset	
Median [IQR]	13.00 [8.00, 20.00]
Time since MS diagnosis	
Median [IQR]	11.50 [6.00, 19.00]
First-line DMT	46 (51.11%)
Interferon	20 (22.22%)
Copaxone	0 (0.0%)
Teriflonomide	6 (6.67%)
Dimethyl fumarate	20 (22.22%)
Second-line DMT	44 (48.89%)
Cladribine	5 (5.56%)
Fingolimod	6 (6.67%)
Alemtuzumab	7 (7.78%)
Natalizumab	8 (8.89%)
Ocrelizumab	11 (12.22%)
Rituximab	7 (7.78%)

* categorical variables expressed as raw frequencies and relative percentages, quantitative variables expressed as median and interquartile range.

**Table 2:** Differences in citokine levels between seropositive (IgG, IgM or IgA) and seronegative patients *.

Cytokines	Seropositive (Median Levels)	Seronegative (Median Levels)	*p*	Rank Biserial Correlation (95% CI)
IL-1	18.29	17.95	0.659	−0.078 (−0.395, 0.255)
IFN-α	8.69	8.25	0.659	−0.254 (−0.536, 0.08)
IL-6	12.82	13.89	0.061	−0.325 (−0.589, 0.002)
IFN-γ	3.92	4.56	0.231	−0.208 (−0.501, 0.127)
TNF-α	5.19	6.96	0.359	−0.16 (−0.462, 0.176)
MCP-1	352.21	432.53	0.091	−0.293 (−0.566, 0.038)
IL-8	41.03	41.57	0.384	−0.152 (−0.456, 0.184)
IL-10	5.51	6.85	0.049	−0.34 (−0.6, −0.015)
IL-12	3.31	3.78	0.174	−0.236 (−0.523, 0.098)
IL-17	1.35	1.58	0.183	−0.231 (−0.531, 0.12)
IL-18	200.22	252.45	0.372	−0.156 (−0.459, 0.18)
IL-23	3.77	7.88	0.025	−0.393 (−0.638, −0.076)
IL-33	21.46	29.67	0.838	−0.036 (−0.362, 0.298)

* Evaluated through Wilcoxon-signed rank test. Rank biserial correlation selected as effect measure.

**Table 3:** Serological Ig-G status at 6 months according with cytokine * levels at basal moment.

Cytokines Median (IQR)	Seropositive	Seronegative	*p* Value
IL-1	16.39 (1.15–51.58)	24.91 (7.64–48.25)	0.5495
IFN-α	6.656 (1.429–21.75)	8.391 (3.476–15.54)	0.8561
IL-6	12.28 (8.15–18.53)	14.43 (9.62–18.55)	0.5585
IFN-γ	5.5 (0.955–9.8)	3.8 (2.125–9.81)	0.783
TNF-α	10.45 (3.12–18.34)	4.90 (2.49–10.01)	0.1609
MCP-1	326 (206.3–871)	390.6 (270.5–498.1)	0.5724
IL-8	48.23 (35.21–72.33)	36.89 (27.30–48.12)	0.0759
IL-10	7.88 (2.37–16.27)	5.37 (1.55–8.18)	0.2062
IL-12	3.54 (1.50–6.17)	3.29 (1.28–4.51)	0.455
IL-17	2.95 (1.67–4.76)	2.16 (1.21–5.63)	0.4884
IL-18	324.60 (208.10–703.70)	177.60 (116.00–320.30)	0.0193 **
IL-23	4.45 (1.13–21.24)	2.90 (0.30–8.71)	0.3222
IL-33	42.41 (3.31–114.40)	20.36 (9.68–65.46)	0.9946

* Expressed as median and interquartile range. ** Statistically significant.

## Data Availability

The original contributions presented in this study are included in the article/Supplementary Material. Further inquiries can be directed to the corresponding author.

## Supplementary Materials

The following supporting information can be downloaded at: https://www.mdpi.com/article/10.3390/jcm14113736/s1, Table S1: Duration of DMTs (months) in general and according with serological status.; Table S2: Mixed logistic regression showing differences in Cytokine Levels in pwMS in Relation to SARS-CoV-2 infection.

## Abbreviations

The following abbreviations are used in this manuscript:

COVID-19	Coronavirus Disease 2019
DMT(s)	Disease-Modifying Therapy(ies)
EDSS	Expanded Disability Status Scale
ELISA	Enzyme-Linked Immunosorbent Assay
FACS	Fluorescence-Activated Cell Sorting
GLMM	Generalized Linear Mixed Models
IQR	Interquartile Range
IFN	Interferon (including IFN-α and IFN-γ)
IL	Interleukin (e.g., IL-1, IL-6, IL-8, IL-10, IL-12, IL-17, IL-18, IL-23, IL-33)
Ig	Immunoglobulin (e.g., IgG, IgM, IgA)
MCP-1	Monocyte Chemoattractant Protein-1
MS	Multiple Sclerosis
NK	Natural Killer (cells)
OR	Odds Ratio
pwMS	Patients with Multiple Sclerosis
RRMS	Relapsing–Remitting Multiple Sclerosis
SPMS	Secondary Progressive Multiple Sclerosis
TNF-α	Tumor Necrosis Factor-alpha
S1/S2	Spike protein subunit 1 and Spike protein subunit 2
5PL	Five-Parameter Logistic curve
