# Supplementary material for: Profile of Cytokines Associated with SARS-CoV2 Seropositivity in Multiple Sclerosis Patients and Its Persistence over Six Months
Source: J Clin Med. 2025 May 26;14(11):3736. doi: 10.3390/jcm14113736 (PMC12155705; doi:10.3390/jcm14113736)
Supplement: Supplementary file 1 [file jcm-14-03736-s001.zip › jcm-3580403-supplementary.pdf]

Supplementary Table S1. Duration of DMTs (months) in general and according with serological status.

**Treatment**	**Serological status**	**Median (IQR)**	**p (Mann-Whitney U test)**
ALEMTUZUMAB	Global	28.0 (27.0–44.0)	
	Negative	27.5 (27.0–32.25)	0,8584
	Positive	43.0 (31.5–47.5)	
TERIFLUNOMIDE	Global	15.5 (3.75–28.0)	
	Negative	16.0 (1.5–30.5)	1
	Positive	15.5 (12.25–18.75)	
anti CD20	Global	6.0 (3.25–12.0)	
	Negative	4.0 (2.0–25.5)	0,7153
	Positive	6.0 (5.0–9.0)	
CLADRIBINE	Global	10.0 (5.0–12.0)	
	Negative	5.0 (4.0–8.5)	0,4
	Positive	14.5 (12.25–16.75)	
FINGOLIMOD	Global	61.0 (45.25–79.75)	
	Negative	45.0 (34.0–63.0)	0,5066
	Positive	76.0 (61.0–78.5)	
INTERFERON	Global	128.0 (70.75–146.0)	
	Negative	142.0 (117.0–155.0)	0,3211
	Positive	114.0 (70.5–141.5)	
NATALIZUMAB	Global	92.5 (46.25–116.0)	
	Negative	82.0 (46.25–120.5)	0,8857
	Positive	92.5 (65.0–107.75)	
DIMETHYL FUMARATE	Global	28.5 (17.0–42.75)	
	Negative	28.5 (21.0–55.5)	0,5887
	Positive	28.0 (9.25–39.25)	

DMT: disease-modifying therapy

Table S2: Mixed logistic regression showing differences in Cytokine Levels in pwMS in Relation to SARS-CoV-2 infection					
**Model**	**Term**	**OR (95%CI)**	**t-statistic**	**p-value**	**Significance**
Model for IL-1	(Intercept)	4.46 (1.077,18.473)	2,062	0,039	p<0.05
	lymphocytes	0.669 (0.364,1.23)	-1,294	0,196	No significance
	IL-1	0.996 (0.811,1.222)	-0,04	0,968	No significance
	Time Since First Symptom Onset (Years)	0.944 (0.892,0.999)	-1,982	0,047	p<0.05
Model for IFN-γ	(Intercept)	15.222 (2.805,82.598)	3,155	0,002	p<0.01
	lymphocytes	0.514 (0.262,1.009)	-1,934	0,053	No significance
	IFN-γ	0.613 (0.414,0.908)	-2,445	0,014	p<0.05
	Time Since First Symptom Onset (Years)	0.934 (0.88,0.992)	-2,226	0,026	p<0.05
Model for IFN-α	(Intercept)	5.66 (1.347,23.784)	2,366	0,018	p<0.05
	lymphocytes	0.641 (0.348,1.179)	-1,431	0,153	No significance
	IFN-α	0.908 (0.716,1.151)	-0,796	0,426	No significance
	Time Since First Symptom Onset (Years)	0.944 (0.891,0.999)	-1,994	0,046	p<0.05
Model for TNF-α	(Intercept)	6.381 (1.451,28.073)	2,452	0,014	p<0.05
	lymphocytes	0.649 (0.356,1.186)	-1,406	0,16	No significance
	TNF-α	0.85 (0.627,1.153)	-1,042	0,297	No significance
	Time Since First Symptom Onset (Years)	0.941 (0.888,0.997)	-2,074	0,038	p<0.05
Model for MCP-1	(Intercept)	75.477 (1.057,5387.84)	1,986	0,047	p<0.05
	lymphocytes	0.696 (0.381,1.27)	-1,181	0,238	No significance
	MCP-1	0.609 (0.301,1.231)	-1,382	0,167	No significance
	Time Since First Symptom Onset (Years)	0.948 (0.895,1.004)	-1,814	0,07	No significance
Model for IL-6	(Intercept)	17.709 (1.986,157.939)	2,574	0,01	p<0.05
	lymphocytes	0.629 (0.34,1.163)	-1,478	0,139	No significance
	IL-6	0.611 (0.334,1.115)	-1,605	0,108	No significance
	Time Since First Symptom Onset (Years)	0.944 (0.89,1)	-1,96	0,05	p<0.05
Model for IL-8	(Intercept)	18.264 (2.162,154.316)	2,668	0,008	p<0.01
	lymphocytes	0.675 (0.368,1.239)	-1,269	0,204	No significance
	IL-8	0.683 (0.438,1.065)	-1,681	0,093	No significance
	Time Since First Symptom Onset (Years)	0.939 (0.886,0.996)	-2,104	0,035	p<0.05
Model for IL-10	(Intercept)	11.185 (2.225,56.231)	2,93	0,003	p<0.01
	Linfos	0.583 (0.308,1.102)	-1,66	0,097	No significance
	IL-10	0.697 (0.495,0.982)	-2,066	0,039	p<0.05
	Time Since First Symptom Onset (Years)	0.933 (0.879,0.991)	-2,251	0,024	p<0.05
Model for IL-12	(Intercept)	8.338 (1.845,37.687)	2,756	0,006	p<0.01
	lymphocytes	0.61 (0.328,1.135)	-1,561	0,119	No significance
	IL-12	0.67 (0.425,1.057)	-1,721	0,085	No significance
	Time Since First Symptom Onset (Years)	0.939 (0.886,0.996)	-2,106	0,035	p<0.05
Model for IL-17	(Intercept)	4.806 (1.307,17.673)	2,363	0,018	p<0.05
	Linfos	0.664 (0.362,1.218)	-1,322	0,186	No significance
	IL-17	0.871 (0.726,1.045)	-1,483	0,138	No significance
	Time Since First Symptom Onset (Years)	0.936 (0.882,0.993)	-2,184	0,029	p<0.05
Model for IL-18	(Intercept)	15.876 (0.459,549.396)	1,529	0,126	No significance
	lymphocytes	0.683 (0.377,1.24)	-1,253	0,21	No significance
	IL-18	0.787 (0.426,1.454)	-0,766	0,444	No significance
	Time Since First Symptom Onset (Years)	0.944 (0.892,1)	-1,97	0,049	p<0.05
Model for IL-23	(Intercept)	7.99 (1.883,33.897)	2,818	0,005	p<0.01
	lymphocytes	0.659 (0.355,1.221)	-1,325	0,185	No significance
	IL-23	0.734 (0.564,0.956)	-2,291	0,022	p<0.05
	Time Since First Symptom Onset (Years)	0.935 (0.881,0.992)	-2,214	0,027	p<0.05
Model for IL-33	(Intercept)	11.946 (1.989,71.74)	2,712	0,007	p<0.01
	lymphocytes	0.598 (0.321,1.117)	-1,612	0,107	No significance
	IL-33	0.779 (0.575,1.056)	-1,61	0,107	No significance
	Time Since First Symptom Onset (Years)	0.941 (0.888,0.997)	-2,055	0,04	p<0.05
